# Supplementary material for: Engineered EVs for Oxidative Stress Protection
Source: Pharmaceuticals (Basel). 2021 Jul 21;14(8):703. doi: 10.3390/ph14080703 (PMC8399368; doi:10.3390/ph14080703)
Supplement: Supplementary file 1 [file pharmaceuticals-14-00703-s001.zip › Suppl Figure 1.pdf]

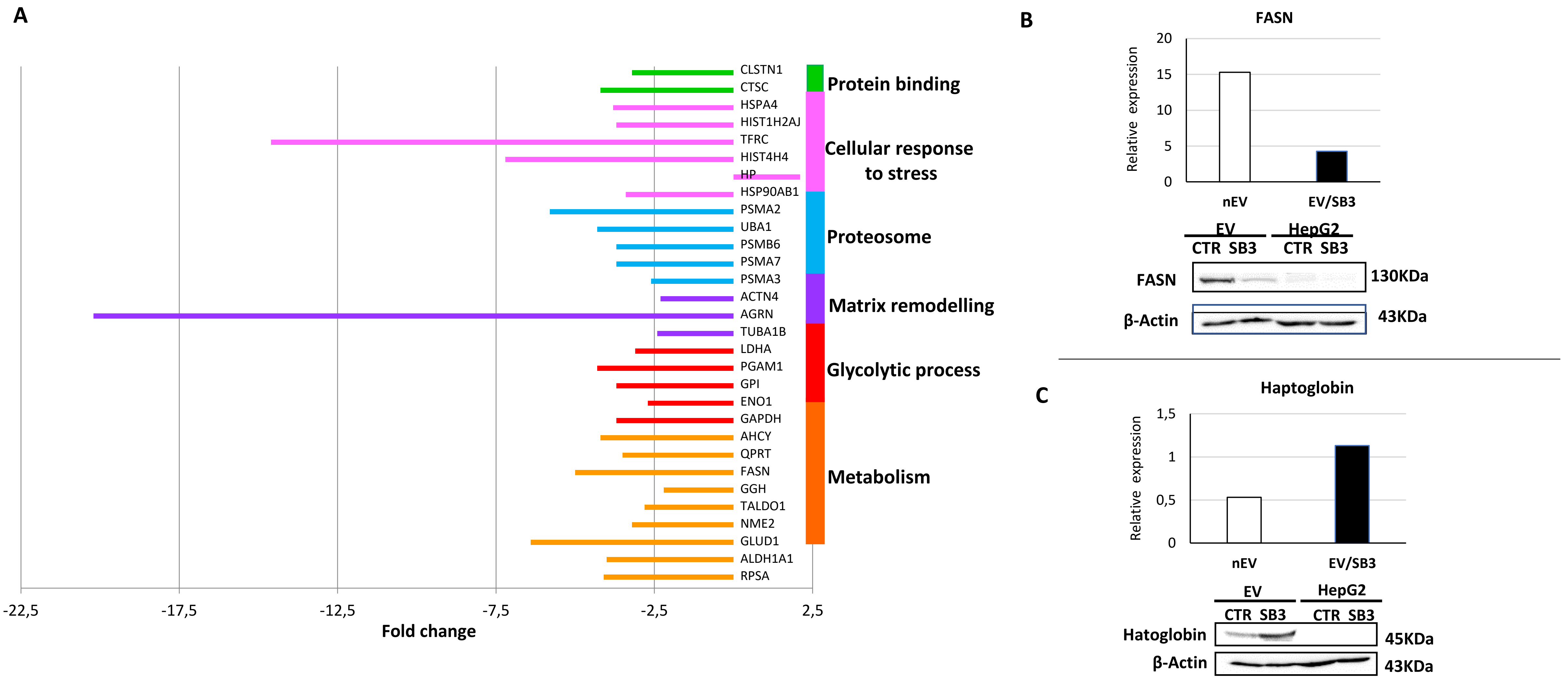

Supplemental Figure 1: **Protein modulation in Evs-SB3 compared to nEVs. A)** Graphical representation of protein modulation in EVs-SB3, in relation to biological function. The results are expressed as fold change compared to the expression of each protein in control EVs. **Right Panels:** Western blot analysis of two proteins differently expressed in EVs-SB3, compared to control EVs, FASN was chosen as underexpressed protein (**B**) and haptoglobin was chosen as over-expressed protein (**C**). Bars represent the densitometric values of Western blot results, normalized by β-actin expression.
